# Genetic Variability of Exotic Sugarcane Genotypes

**DOI:** 10.1155/2017/5202913

**Published:** 2017-12-03

**Authors:** M. N. Alam, Ujjal Kumar Nath, K. M. R. Karim, M. M. Ahmed, R. Y. Mitul

**Affiliations:** ^1^Bangladesh Sugarcrop Research Institute, Regional Station, Gazipur 1701, Bangladesh; ^2^Department of Genetics and Plant Breeding, Bangladesh Agricultural University, Mymensingh 2200, Bangladesh; ^3^Bangladesh Sugarcrop Research Institute, Ishurdi, Pabna 6620, Bangladesh

## Abstract

Sugarcane is the main sugar producing crop in Bangladesh. However, improvement of this crop through breeding is limited due to lack of genetic diversity. Therefore, genetic variability and diversity assessment are necessarily important for the foreign introduced materials. Experiment was conducted with 9 exotic sugarcane genotypes at Regional Station, Bangladesh Sugar Crop Research Institute, Gazipur, during 2012-13, following RCBD. Data were collected on different growth and yield contributing characters. Individual cane weight exhibited high genotypic coefficient of variation and phenotypic coefficient of variation. Leaf blade length, leaf blade width, fresh leaf weight, dried leaf weight, number of tillers, millable cane, bud size, cane diameter, internodes number, internode length, plant height, stalk length, brix%, and individual cane weight showed high heritability. Individual cane weight showed highly significant and positive correlation with cane diameter, internode length, and stalk length, whereas path coefficient analysis revealed that cane diameter had maximum positive direct effect on individual cane weight followed by internode length, number of tillers, and chlorophyll content. Results indicate that the genotypes should be selected on the basis of individual cane weight, cane diameter, and millable canes number for future breeding to get higher sugarcane yield in respect to juice and brix content.

## 1. Introduction

Sugarcane is the leading sugar producing crop in the world as well as in Bangladesh. It provides about 75% of the sugar harvested for human consumption [1, 2]. The average yield of sugarcane in Bangladesh is about 41.2 tons/hectare which is far below from the existing standard; therefore, possibilities could be exploited through collaboration among research stations and progressive growers [3]. Sugarcane is a long duration field crop which occupies the land up to 12–18 months for its maturity. It is considered as a time-consuming crop compared to other traditional field crops grown in Bangladesh. Therefore, sustainability of sugarcane cultivation in this country is threatened. To sustain sugarcane production and to improve the productivity, tolerance to biotic and abiotic stresses, nutrient management, and improved sugar recovery are of the major concerns. Development of varieties is important consideration that would be highly productive and tolerant against biotic and abiotic factors with the changing climate. Coefficients of variation with heritability as well as genetic advance are very essential to improve any trait of sugarcane because this would help in informing whether or not the desired objective can be achieved from the material [4]. Therefore, the objective of present study was to narrate the nature and extent of genetic variability and phenotypic and genotypic variability of sugarcane varieties in some exotic traits in Bangladesh.

## 2. Materials and Methods

The experiment was conducted at Regional Station, Bangladesh Sugar crop Research Institute, Gazipur, during 2012-13 cropping season under Madhupur Tract soil, following Randomized Completely Block Design (RCBD) with three replicates. Nine exotic genotypes of sugarcane, namely, GT11, GT15, GT17, VMC86-550, HoCP85-845, HoCP96-540, HoCP95-988, HoCP91-555, and CB45-3, were collected from Quarantine Station, Bangladesh Sugar Crop Research Institute, Gazipur. The two-eyed setts of each genotype were planted in 6 m × 5 m size plot. Line to line distance was 1 m and plot to plot was 2 m. Setts were placed in the furrow following end to end method. Data were collected on different growth and yield contributing characters. Intercultural operations like weeding, earthen-up, mulching, and irrigation were done as per required schedule. Leaf chlorophyll content (SPAD index) was estimated using a SPAD-502 plus chlorophyll meter [5]. The collected data were analyzed by different statistical software, namely. MSTAT-C [6], PLABSTAT, and STAR [7] program for variability and diversity analysis. Analysis of variance was performed using the Plant Breeding Statistical Program [8].

### 2.1. Estimation of Genotypic and Phenotypic Variances

Genotypic and phenotypic variances were calculated using the following formula [9, 10]:

(1) $$\begin{array}{c} Genotypic\;\;variance\;\;\left(\;\sigma_{g}^{2}\;\right)=\frac{GMS-EMS}{r}, \end{array}$$

where GMS is genotypic mean square, EMS is error mean square, *r* is number of replication, and phenotypic variance is (*σ*_*p*_^2^) = *σ*_*g*_^2^ + *σ*_*e*_^2^.

### 2.2. Estimation of Genotypic Coefficient of Variation (GCV) and Phenotypic Coefficient of Variation (PCV)

Phenotypic (PCV) and genotypic (GCV) coefficients of variation were evaluated according to the methods as follows [10–13]:

(2) Genotypic  coefficient  of  variation  GCV=σg2X−×100,

where *σ*_*g*_^2^ is genotypic variance and $\overset{-}{X}$ is population mean.

(3) Phenotypic  coefficient  of  variation  PCV=σp2X−×100,

where *σ*_*p*_^2^ is phenotypic variance and $\overset{-}{X}$ is population mean.

### 2.3. Estimation of Heritability

Broad-sense heritability (*h*^2^) for mean values was calculated using PABSTAT [8], following the formula described by [9, 10, 14, 15]:

(4) $$\begin{array}{c} Heritability\;\;\left(\;h_{b}^{2}\;\right)=\frac{\sigma_{g}^{2}}{\sigma_{p}^{2}}\times 100, \end{array}$$

where *σ*_*g*_^2^ is genotypic variance and *σ*_*p*_^2^ is phenotypic variance.

### 2.4. Estimation of Genetic Advance

Genetic advance (GA) was estimated accordance to the methods illustrated [10, 16, 17]:

(5) $$\begin{array}{c} Genetic\;\;advance\;\;\left(\;GA\;\right)=h_{b}^{2}\cdot K\cdot \sigma_{p}, \end{array}$$

where *h*_*b*_^2^ is heritability in broad sense, *K* = *K* is the selection differential value which is 2.06 at 5% selection intensity, and *σ*_*p*_ is phenotypic standard deviation.

### 2.5. Estimation of Correlation Coefficient

The genotypic and phenotypic correlation coefficients between growth and yield contributing character were calculated as follows [13]:

(6) $$\begin{array}{c} Genotypic\;\;correlation,r_{g\left(xy\right)}=\frac{{Cov}_{\left(g\right)1.2}}{\sqrt{\sigma^{2_{\left(g\right)1}}\sigma^{2_{\left(g\right)2}}}}. \end{array}$$

Cov_(*g*)1.2_ is genotypic covariance between the variables *X* and *Y*, *σ*^2_(*g*)1_^ is genotypic variance of the variable *X*_1_, and *σ*^2_(*g*)2_^ is genotypic variance of the variable *X*_2_.

(7) $$\begin{array}{c} Phenotypic\;\;correlation,r_{p\left(xy\right)}=\frac{{Cov}_{\left(p\right)1.2}}{\sqrt{\sigma^{2_{\left(p\right)1}}\sigma^{2_{\left(p\right)2}}}}. \end{array}$$

Cov_(*p*)1.2_ is phenotypic covariance between the variables *X* and *Y*, *σ*^2_(*p*)1_^ is phenotypic variance of the variable *X*_1_, and *σ*^2_(*p*)2_^ is Phenotypic variance of the variable *X*_2_.

### 2.6. Estimation of Path Coefficient

Direct and indirect path coefficient was calculated as described [18]:

(8) ryi=Pyi+∑i=1i′=1krii′Pyi′ for  i≠1,

where *r*_*yi*_ is the correlation coefficient between the *i*th causal variable (*Xi*) and effect variable (*y*), *r*_*ii*′_ is the correlation coefficient between the *i*th and *i*′th causal variables, *P*_*yi*_ is the path coefficient (direct effect) of the *i*th causal variable (*Xi*), and *r*_*ii*′_*P*_*yi*′_ is the indirect effect of the *i*th causal variable via the *i*′th causal variable. To determine the direct effect, square matrices of the correlation coefficients between independent traits in all possible pairs were inverted and multiplied by the correlation coefficient between the independent and dependent traits.

## 3. Results and Discussion

### 3.1. Variance Components

The analysis of variance for all characters showed statistically highly significant (*p* ≤ 0.01) among the genotypes except chlorophyll (Table 1). Similar results were also found in case of number of millable canes, individual cane weight, cane height, and sucrose% [19]. These results indicated that there were greater variations among the exotic genotypes that might support the design of a breeding program for sugarcane improvement. As stated, the PCV (phenotypic coefficient of variation) and GCV (genotypic coefficient of variation) values are ranked as low, medium, and high with 0 to 10%, 10 to 20%, and >20%, respectively [20]. High GCV were recorded for fresh leaf weight (22.51), millable cane (22.28), bud size (24.02), and individual cane weight (37.79); while leaf blade width (19.43), dried leaf weight (15.42), number of tillers (16.20), and cane diameter (17.58) showed medium GCV and leaf blade length (4.45), chlorophyll content (5.39), number of internodes (5.00), internode length (8.55), plant height (7.14), stalk length (4.38), and brix% (7.05) exhibited low GCV. High phenotypic coefficients of variation (PCV) were also recorded for leaf blade width (20.31), fresh leaf weight (22.78), millable cane (23.19), bud size (24.87), and individual cane weight (37.96) but moderate PCVs were recorded for dried leaf weight (18.26), number of tillers (17.64), cane diameter (18.16), and chlorophyll content (11.88); in contrast, remaining traits showed low PCV (Table 2). High genotypic coefficient of variation (37.79) and phenotypic coefficient of variation (37.96) were found in individual cane weight [21]. The estimated phenotypic coefficient of variation (PCV) was higher than genotypic coefficient of variation (GCV) for all the traits indicating greater environmental influence on these traits for total variation. High GCV and PCV indicated that selection may be effective based on these characters and their phenotypic expression would be good indication of the genotypic potential [22]. Mean performance of different genotypes had wider variation in performance values for different traits (Table 3).

### 3.2. Heritability and Genetic Advance

Heritability values are categorized as low (0–30%), moderate (30–60%), and high (60% and above) [23]. The characters of leaf blade length, leaf blade width, fresh leaf weight, dried leaf weight, number of tillers, millable cane, bud size, cane diameter, number of internodes, internode length, plant height, stalk length, brix%, and individual cane weight showed high heritability except chlorophyll content (45.3%) (Table 2). The heritability for millable canes number (88%), stalk diameter (85%), and cane weight (84%) were also reported in sugarcane [19]. Similar results were found for those characters [16, 22]. It indicates that simple selection based on phenotype for these traits might be effective method for sugarcane variety improvement breeding program. The highest genetic advance was found in millable cane (10.645) and the lowest in stalk length (0.005; Table 2).

### 3.3. Correlation Coefficient

The pairwise simple correlation coefficient (*r*) among various variables of nine exotic genotypes is presented in Table 4. Individual cane weight showed positive and highly significant correlation with cane diameter (*r* = 0.942^*∗∗*^), internode length (*r* = 0.837^*∗∗*^), and stalk length (*r* = 0.775^*∗*^). There was also positive significant correlation of individual cane weight with leaf blade width (*r* = 0.784^*∗*^), fresh leaf weight (*r* = 0.807^*∗∗*^), dried leaf weight (*r* = 0.765^*∗*^), nonsignificant positive correlation with leaf blade length (*r* = 0.453), bud size (*r* = 0.078), chlorophyll content (*r* = 0.014), number of internodes (*r* = 0.523), plant height (*r* = 0.522), and brix% (*r* = 0.482). By contrast, number of tillers (*r* = −0.721^*∗*^) and millable cane (*r* = −0.707^*∗*^) had negative significant correlations with individual cane weight. Positive and highly significant correlation between cane yield and its components, namely, single cane weight, stalk length, and millable canes number, was reported [24–26]. It was also observed that cane diameter has significant positive correlation with cane yield [27]. Millable canes number had negatively significant correlation with cane diameter (*r* = −0.722^*∗*^), internode length (*r* = −0.676^*∗*^), and brix% (*r* = −0.742^*∗*^). It was also reported that millable canes number had negative significant correlation with cane diameter (*r* = −0.722^*∗*^) [24]. It is obvious that single cane weight, stalk length, millable canes number, stalk diameter, and number of internodes can be considered together in a positive direction towards an ultimate aim of developing high yielding sugarcane clone.

### 3.4. Path Coefficient Analysis

Path coefficient analysis was performed to partition the correlation coefficient value towards individual cane weight into direct and indirect effect to get the real scenario of that trait into target variable. The results of path coefficient analysis revealed that cane diameter had maximum positive direct effect on individual cane weight (0.748) followed by internode length (0.676), number of tillers (0.410), chlorophyll (0.308), dried leaf weight (0.272), leaf blade length (0.229), and number of internodes (0.188) (Table 5). Path coefficient analyses indicated that plant height was less important contributors than stalk diameter and stalk number for enhancing cane yield [28]. It was reported that numbers of internodes were the major contributors to cane yield per plot [29]. This study indicates that cane diameter, number of internodes, length of internode, and stalk length were most important for getting higher individual cane weight as well as improvement of sugarcane yield. Therefore, selection based on number of millable canes and single cane weight might directly increase sugarcane yield.

### 3.5. Divergence of Genotypes

**Figure 1:**
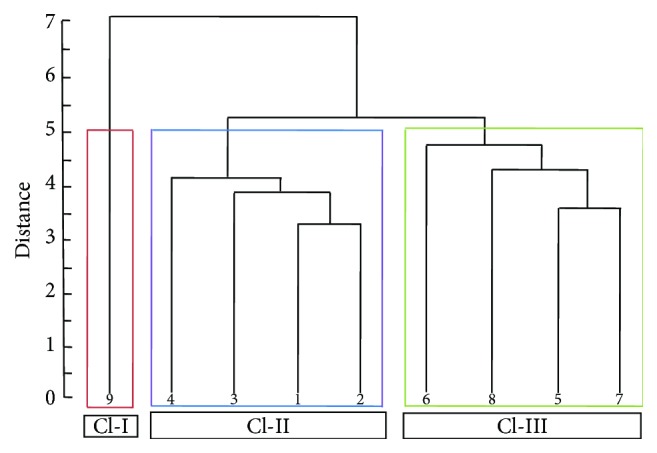
Dendrogram based on mean performance of variables among 9 exotic sugarcane genotypes according to average clustering and Euclidean distance method, where 1 is GT11, 2 is GT15, 3 is GT17, 4 is VMC86-550, 5 is HoCP85-845, 6 is HoCP96-540, 7 is HoCP95-988, 8 is HoCP91-555, and 9 is CB45-3.

All the genotypes were clustered on the basis of agglomerative cluster analysis, where specifications were made based on Euclidean distance matrix (Table 6) and grouping was made on average clustering method. Based on these two methods together the nine genotypes were clustered into three groups named as cluster I, cluster II, and cluster III (Figure 1). Cluster II included 4 genotypes (GT 11, GT 15, GT 17, and VMC 86-550). Similarly, cluster III also included 4 genotypes (HoCP85-845, HoCP95-988, HoCP91-555, and HoCP96-540). By contrast, only genotype CB45-3 belonged to cluster I.

## 4. Conclusion

The study indicated that there is wide genetic variability among the tested genotypes for growth and yield characters. Moreover, the results showed high GCV for millable cane (22.28) and individual cane weight (37.79), while leaf blade length (4.45), chlorophyll content (5.39), number of internodes (5.00), internode length (8.55), plant height (7.14), stalk length (4.38), and brix% (7.05) showed low GCV. High phenotypic coefficient of variation was also recorded for millable cane (23.19) and individual cane weight (37.96). Path coefficient value in plant height is less important than stalk diameter and stalk number as a component of cane yield. Therefore, path coefficient, GCV, and PCV together might be helpful for effective selection. However, selection of candidate genotypes should also be performed considering those characters with high values of heritability because they magnify the genetic advance to progenies.

## Figures and Tables

**Table 1:** Analysis of variances for 15 characters of 9 exotic sugarcane genotypes.

Sources	df	LBL	LBW	FLW	DLW	NT	MC	Bud size	Chlorophyll content	Cane diameter	Number of internodes	Internode length	Plant height	Stalk length	Brix%	ICW
Genotype	8	125.15	1.45	1906.43	84.37	12.34	14.91	252.24	46.67	0.42	8.04	2.71	0.32	0.19	6.00	0.72
Replication	2	2.81	0.01	5.15	29.48	1.59	2.01	13.70	0.62	0.01	1.93	0.81	0.12	0.02	2.02	0.00
Error	16	4.95	0.04	15.19	9.98	0.72	0.41	5.94	26.25	0.01	1.30	0.06	0.02	0.01	0.37	0.00

*Note*. LBL = leaf blade length, LBW = leaf blade width, FLW = fresh leaf weight, DLW = dried leaf weight, NT = number of tillers, MC = millable cane, BS = bud size, chlorophyll, CD = cane diameter, NI = number of internodes, IL = internode length, PH = plant height, SL = stalk length, brix%, and ICW = individual cane weight.

**Table 2:** Component for variances, heritability in broad sense (*h*_*b*_^2^), and genetic advance (GA) for 15 variables of 9 exotic sugarcane genotypes.

Characters	GV	EV	PV	GCV	PCV	Heritability (*h*_*b*_^2^)	GA
Leaf blade length	40.07	4.947	45.02	4.45	4.71	94.3	61.456
Leaf blade width	0.47	0.043	0.51	19.43	20.31	95.7	28.618
Fresh leaf weight	630.41	15.190	645.60	22.51	22.78	98.8	1178.444
Dried leaf weight	24.80	9.982	34.78	15.42	18.26	84.4	187.288
Number of tillers	3.88	0.718	4.59	16.20	17.64	91.9	71.525
Millable cane	4.84	0.405	5.24	22.28	23.19	96.1	105.069
Bud size	82.10	5.935	88.04	24.02	24.87	96.6	464.190
Chlorophyll	6.80	26.253	33.06	5.39	11.88	45.4	63.850
Cane diameter	0.14	0.009	0.15	17.58	18.16	96.8	13.873
Number of internodes	2.25	1.301	3.55	5.00	6.29	79.6	19.402
Internode length	0.88	0.061	0.94	8.55	8.84	96.7	17.106
Plant height	0.10	0.017	0.12	7.14	7.69	92.8	5.075
Stalk length	0.02	0.011	0.03	4.38	5.63	77.9	1.489
Brix%	1.88	0.370	2.25	7.05	7.71	91.4	21.757
Individual cane weight	0.24	0.002	0.24	37.79	37.96	99.6	38.115

*Note*. GV = genotypic variance, EV = error variance, PV = phenotypic variance, GCV = genotypic coefficient of variation, and PCV = phenotypic coefficient of variation.

**Table 3:** Mean performance of the exotic nine genotypes for 15 different variables.

Variables	Genotypes									
	GT 11	GT 15	GT 17	VMC 86-550	HoCP 85-845	HoCP 96-540	HoCP 95-988	HoCP 91-555	CB 45-3	±LSD_(0.05)_
Leaf blade length (cm)	143.28	143.71	150.35	143.66	133.28	132.03	140.53	150.82	143.60	3.85
Leaf blade width (cm)	4.56	4.31	3.87	3.62	3.69	3.16	3.0	3.12	2.33	0.36
Fresh leaf weight (g)	136.67	133.33	128.67	144.33	106.00	89.33	85.33	105.33	74.67	6.75
Dried leaf weight (g)	38.67	38.00	37.33	36.00	31.33	25.33	28.0	30.00	26.00	5.47
Number of tillers/m^2^	9.67	10.33	13.00	11.67	12.00	10.33	13.33	12.67	16.33	1.47
Millable cane/m^2^	8.00	7.33	10.33	9.67	9.67	7.67	11.0	10.50	14.67	1.10
Bud size (mm^2^)	40.50	53.84	29.82	46.58	39.33	36.20	32.05	22.68	38.58	4.22
Chlorophyll (spad)	48.67	45.03	46.07	49.23	49.53	57.40	43.97	49.23	46.33	8.87
Cane diameter (cm)	2.64	2.35	2.45	2.29	1.89	1.96	2.0	2.07	1.36	0.17
Number of internodes	30.00	33.00	29.00	28.67	29.33	30.00	29.0	32.33	28.33	1.97
Internode length (cm)	12.24	12.19	11.47	11.06	9.44	11.34	10.64	10.57	9.92	0.43
Plant height (m)	4.82	4.76	4.93	3.90	4.40	4.61	4.45	4.41	4.16	0.22
Stalk length (m)	3.13	3.05	3.14	3.15	2.38	3.01	2.76	2.99	2.77	0.18
Brix%	19.83	19.38	19.02	19.77	20.22	20.54	20.41	19.83	15.88	1.05
Individual cane weight (kg)	1.88	1.68	1.64	1.48	0.78	1.2	1.0	1.57	0.39	0.08

**Table 4:** Correlation coefficient matrix among different characters in 9 exotic sugarcane genotypes.

Variables	Leaf blade length	Leaf blade width	Fresh leaf weight	Dried leaf weight	Number of tillers	Millable cane	Bud size	Chlorophyll content	Cane diameter	Number of internodes	Internode length	Plant height	Stalk length	Brix%
Leaf blade width	0.090													
Fresh leaf weight	0.354	0.856^*∗∗*^												
Dried leaf weight	0.438	0.892^*∗∗*^	0.945^*∗∗*^											
Number of tillers	0.276	−0.795^*∗*^	−0.613	−0.506										
Millable cane	0.279	−0.777^*∗*^	−0.581	−0.484	0.981^*∗∗*^									
Bud size	−0.300	0.442	0.439	0.425	−0.373	−0.369								
Chlorophyll content	−0.552	−0.080	−0.123	−0.357	−0.421	−0.393	−0.099							
Cane diameter	0.322	0.891^*∗∗*^	0.865^*∗∗*^	0.856^*∗∗*^	−0.741^*∗*^	−0.722^*∗*^	0.171	−0.077						
Number of internodes	0.235	0.370	0.247	0.248	−0.471	−0.525	0.104	0.004	0.315					
Internode length	0.264	0.693^*∗*^	0.627	0.639	−0.676^*∗*^	−0.674^*∗*^	0.345	0.001	0.795^*∗*^	0.420				
Plant height	0.122	0.570	0.212	0.387	−0.433	−0.494	−0.108	−0.060	0.546	0.378	0.598			
Stalk length	0.540	0.377	0.579	0.500	−0.378	−0.359	0.088	0.073	0.646	0.242	0.831^*∗∗*^	0.267		
Brix%	−0.329	0.455	0.304	0.170	−0.742^*∗*^	−0.759^*∗*^	−0.081	0.352	0.543	0.278	0.251	0.239	0.054	
Individual cane weight	0.453	0.784^*∗*^	0.807^*∗∗*^	0.765^*∗*^	−0.721^*∗*^	−0.707^*∗*^	0.078	0.014	0.942^*∗∗*^	0.523	0.837^*∗∗*^	0.522	0.775^*∗*^	0.482

^*∗*, *∗∗*^Significant at 5% and 1%, respectively.

**Table 5:** Path coefficient analysis showing direct (diagonal) and indirect effects of different characters on individual cane weight of sugarcane genotypes.

Characters	LBL	LBW	FLW	DLW	NT	MC	BS	Chlorophyll	CD	NI	IL	PH	SL	Brix
LBW	0.229	−0.007	−0.037	0.119	0.113	−0.102	0.068	−0.170	0.241	0.044	0.179	−0.040	−0.203	0.017
FLW	0.021	−0.075	−0.090	0.243	−0.327	0.283	−0.101	−0.025	0.667	0.070	0.469	−0.186	−0.141	−0.024
DLW	0.081	−0.065	−0.104	0.257	−0.252	0.212	−0.100	−0.038	0.648	0.047	0.424	−0.069	−0.217	−0.016
NT	0.100	−0.067	−0.099	0.272	−0.208	0.176	−0.097	−0.110	0.641	0.047	0.432	−0.126	−0.188	−0.009
MC	0.063	0.060	0.064	−0.138	0.410	−0.357	0.085	−0.130	−0.555	−0.089	−0.457	0.141	0.142	0.039
BS	0.064	0.059	0.061	−0.132	0.403	−0.364	0.084	−0.121	−0.541	−0.099	−0.456	0.161	0.135	0.040
Chlorophyll	−0.069	−0.033	−0.046	0.116	−0.153	0.134	−0.228	−0.030	0.128	0.020	0.233	0.035	−0.033	0.004
CD	−0.126	0.006	0.013	−0.097	−0.173	0.143	0.023	0.308	−0.058	0.001	0.001	0.020	−0.027	−0.018
NI	0.074	−0.067	−0.090	0.233	−0.305	0.263	−0.039	−0.024	0.748	0.060	0.538	−0.178	−0.242	−0.028
IL	0.054	−0.028	−0.026	0.067	−0.194	0.191	−0.024	0.001	0.236	0.188	0.284	−0.123	−0.091	−0.014
PH	0.060	−0.052	−0.066	0.174	−0.278	0.246	−0.079	0.000	0.595	0.079	0.676	−0.195	−0.312	−0.013
SL	0.028	−0.043	−0.022	0.105	−0.178	0.180	0.025	−0.018	0.409	0.071	0.405	−0.326	−0.100	−0.012
Brix	0.123	−0.028	−0.061	0.136	−0.155	0.131	−0.020	0.022	0.484	0.046	0.562	−0.087	−0.375	−0.003
ICW	−0.075	−0.034	−0.032	0.046	−0.305	0.277	0.018	0.108	0.407	0.053	0.170	−0.078	−0.020	−0.052
Residual effect = 0.019														

*Note*. LBL = leaf blade length, LBW = leaf blade width, FLW = fresh leaf weight, DLW = dried leaf weight, NT = number of tillers, MC = millable cane, BS = bud size, chlorophyll, CD = cane diameter, NI = number of internodes, IL = internode length, PH = plant height, SL = stalk length, brix%, and ICW = individual cane weight.

**Table 6:** Euclidean distance matrix for 15 characters of 9 exotic sugarcane genotypes.

Genotypes	GT 11	GT 15	GT 17	VMC 86-550	HoCP 85-845	HoCP 96-540	HoCP 95-988	HoCP 91-555	CB 45-3
GT 11	0	2.773618	3.147112	3.972764	6.141488	5.404492	5.806524	5.1793	9.16596
GT 15	2.773618	0	4.367265	4.508293	6.214665	5.954076	5.983032	5.355682	8.923348
GT 17	3.147112	4.367265	0	4.097314	5.794336	5.814613	4.434417	3.806455	7.27551
VMC 86-550	3.972764	4.508293	4.097314	0	4.92496	5.140553	4.613214	4.588661	7.020552
HoCP 85-845	6.141488	6.214665	5.794336	4.92496	0	4.417262	3.307413	5.016917	5.896278
HoCP 96-540	5.404492	5.954076	5.814613	5.140553	4.417262	0	4.538716	4.798698	7.331457
HoCP 95-988	5.806524	5.983032	4.434417	4.613214	3.307413	4.538716	0	3.588104	4.84463
HoCP 91-555	5.1793	5.355682	3.806455	4.588661	5.016917	4.798698	3.588104	0	6.315618
CB 45-3	9.16596	8.923348	7.27551	7.020552	5.896278	7.331457	4.84463	6.315618	0
